# Silibinin induces apoptosis via calpain-dependent AIF nuclear translocation in U87MG human glioma cell death

**DOI:** 10.1186/1756-9966-30-44

**Published:** 2011-04-19

**Authors:** Ji C Jeong, Won Y Shin, Thae H Kim, Chae H Kwon, Jae H Kim, Yong K Kim, Ki H Kim

**Affiliations:** 1 Department of Oriental Medicine, Dongguk University, Kyung Ju, 780-714, Korea; 2 Department of Physiology, College of Medicine, Pusan National University, Yangsan, Gyeongsangnam-do, 626-770, Korea; 3 Department of Obstetrics and Gynecology, College of Medicine, Pusan National University, and Medical Research Institute and Pusan Cancer Center, Pusan National University Hospital, Pusan, 602-739, Korea

## Abstract

**Background:**

Silibinin, a natural polyphenolic flavonoid, has been reported to induce cell death in various cancer cell types. However, the molecular mechanism is not clearly defined. Our previous study showed that silibinin induces glioma cell death and its effect was effectively prevented by calpain inhibitor. The present study was therefore undertaken to examine the role of calpain in the silibinin-induced glioma cell death.

**Methods:**

U87MG cells were grown on well tissue culture plates and cell viability was measured by MTT assay. ROS generation and △ψ_m _were estimated using the fluorescence dyes. PKC activation and Bax expression were measured by Western blot analysis. AIF nuclear translocation was determined by Western blot and immunocytochemistry.

**Results:**

Silibinin induced activation of calpain, which was blocked by EGTA and the calpain inhibitor Z-Leu-Leu-CHO. Silibinin caused ROS generation and its effect was inhibited by calpain inhibitor, the general PKC inhibitor GF 109203X, the specific PKC_δ _inhibitor rottlerin, and catalase. Silibinin-induce cell death was blocked by calpain inhibitor and PKC inhibitors. Silibinin-induced PKC_δ _activation and disruption of △ψ_m _were prevented by the calpain inhibitor. Silibinin induced AIF nuclear translocation and its effect was prevented by calpain inhibitor. Transfection of vector expressing microRNA of AIF prevented the silibinin-induced cell death.

**Conclusions:**

Silibinin induces apoptotic cell death through a calpain-dependent mechanism involving PKC, ROS, and AIF nuclear translocation in U87MG human glioma cells.

## Background

Glioblastoma is the most lethal and frequent primary brain tumors [1]. It is comprised of poorly differentiated heterogeneous neoplastic astrocytes with aggressive proliferation and highly invasive properties. After diagnosis of glioblastoma, the median survival time of 9-12 months has remained unchanged despite aggressive treatment including surgery, radiation, and chemotherapy [2,3]. Thus, new effective strategies for controlling glioblastoma are required. Because glioblastoma cells avoid differentiation and apoptosis, the induction of differentiation and apoptosis in glioblastoma cells may be considered as a potential treatment strategy.

Silibinin, a natural polyphenolic flavonoid, is a major bioactive component of silymarin which is isolated from the plant milk thistle (*Silybum marianum*), and has been extensively used for its hepatoprotective effects in Asia and Europe. It has been reported that silibinin has anticancer activities in various cancers including prostate cancer in both *in vitro *and *in vivo *models [4-7]. Recently, we observed that silibinin induces apoptosis through Ca^2+^/ROS-dependent mechanism in human glioma cells [8]. The study showed that silibinin-induced cell death was prevented by calpain inhibitor, suggesting involvement of calpain activation in apoptosis induced by silibinin. Therefore, the present study was undertaken to examine role of calpain in the sililbinin-induced glioma cell death. The present study demonstrated that silibinin induces human glioma cell death via a calpain-dependent AIF nuclear translocation involving ROS and PKC.

## Materials and methods

### Reagents

Silibinin, GF 109203X, rottlerin, catalase, MTT, propidium iodide was purchased from Sigma-Aldrich Chemical (St. Louis, MO, USA). Z-Leu-Leu-CHO was purchased from BIOMOL International LP (Plymouth Meeting, PA, USA). DCFH-DA and DiOC_6_(3) were obtained from Molecular Probes (Eugene, OR, USA). Antibodies were obtained from Cell Signaling Technology Inc. (Beverly, MA, USA). All other chemicals were of the highest commercial grade available.

### Cell culture

U87MG cells were obtained from the American Type Culture Collection (Rockville, MD, USA) and maintained by serial passages in 75-cm^2 ^culture flasks (Costar, Cambridge, MA, USA). The cells were grown in Dulbecco's modified Eagle's medium (DMEM, Gibco BRL, Invitrogen, Carsbad, CA, USA) containing 10% heat inactivated fetal bovine serum (HyClone, Logan, UT, USA) at 37°C in humidified 95% air/5% CO_2 _incubator. When the cultures reached confluence, subculture was prepared using a 0.02% EDTA-0.05% trypsin solution. The cells were grown on well tissue culture plates and used 1-2 days after plating when a confluent monolayer culture was achieved. Unless otherwise stated, cells were treated with silibinin in serum-free medium. Test reagents were added to the medium 30 min before silibinin exposure.

### Measurement of cell viability

Cell viability was evaluated using a MTT assay [9]. Culture medium containing 0.5 mg/ml of MTT was added to each well. The cells were incubated for 2 h at 37°C, the supernatant was removed and the formed formazan crystals in viable cells were solubilized with 0.11 ml of dimethyl sulfoxide. A 0.1 ml aliquot of each sample was then translated to 96-well plates and the absorbance of each well was measured at 550 nm with ELISA Reader (FLUOstar OPTIMA, BMG LABTECH, Offenburg, Germany). Data were expressed as a percentage of control measured in the absence of silibinin.

### Measurement of calpain activity

Calpain activity was measured by calpain assay kit (BioVision Research Products, CA, USA) according to the manufacturer's instructions. Cells were grown in 6-well plates and were treated as indicated. Detached cells from the bottom of culture plates by trypsin were pelleted by centrifugation and washed with phosphate-buffered saline (PBS). The pellet were suspended in extraction buffer and incubated on ice for 20 min then centrifuged at 10,000 × g for 10 min at 4°C. The supernatant represented the cytosolic protein. Add 10 μl of 10× reaction buffer and 5 μl of calpain substrate, Ac-LLY-AFC, to each assay. Incubate at 37°C for 1 h in the dark. After incubation, production of free AFC was fluorometrically measured suing a Victor 3 Multilabel Counter with an excitation filter of 400 nm and an emission filter of 505 nm (PerkinElmer, Boston, MA, USA).

### Measurement of reactive oxygen species (ROS)

The intracellular generation of ROS was measured using DCFH-DA. The nonfluorescent ester penetrates into the cells and is hydrolyzed to DCFH by the cellular esterases. The probe (DCFH) is rapidly oxidized to the highly fluorescent compound DCF in the presence of cellular peroxidase and ROS such as hydrogen peroxide or fatty acid peroxides. Cells cultured in 24-well plate were preincubated in the culture medium with 30 μM DCFH-DA for 1 h at 37°C. After the preincubation, the cells were exposed to 30 μM silibinin for various times. Changes in DCF fluorescence was assayed using FACSort Becton Dickinson Flow Cytometer (Becton-Dickinson Bioscience, San Jose, CA, USA) and data were analyzed with CELLQuest Software.

### Measurement of △ψ_m_

The △ψ_m _was measured with DiOC_6_(3), a fluorochrome that is incorporated into cells depending upon the mitochondrial membrane potential [10]. Loss in DiOC_6_(3) staining indicates disruption of the △ψ_m_. Cells were stained with DiOC_6_(3) at a final concentration of 50 nM for 20 min at 37°C in the dark. Cells were washed and resuspended in Hank's balanced salts solution containing Ca^2+ ^and Mg^2+^. The fluorescence intensity was analyzed with a FACScan flow cytometer using the fluorescence signal 1 channel.

### Western blot analysis

Cells were harvest at various times after silibinin treatment and disrupted in lysis buffer (1% Triton X-100, 1 mM EGTA, 1 mM EDTA, 10 mM Tris-HCl, pH 7.4). Cell debris was removed by centrifugation at 10,000 g for 10 min at 4°C. The resulting supernatants were resolved on a 10% SDS-PAGE under denatured reducing conditions and transferred to nitrocellulose membranes. The membranes were blocked with 5% non-fat dried milk at room temperature for 30 min and incubated with different primary antibodies. The membranes were washed and incubated with horseradish peroxidase-conjugated secondary antibodies. The signal was visualized using an enhanced chemiluminescence (Amersham, Buckinghamshire, UK).

### Measurement of AIF nuclear translocation

Cells were harvested and washed twice with PBS. The cells were incubated with extraction buffer (10 mM Hepes, 250 mM sucrose, 10 mM KCl, 1.5 mM MgCl_**2**_, 1 mM EDTA, 1 mM EGTA, 0.05% digitonin, and 1 mM phenylmethylsulfonyl fluoride) at 4°C for 10 min, then centrifuged at 100000 *g *for 10 min at 4°C. The supernatant cytosolic protein was removed and the pellet was incubated in the nuclear extraction buffer (350 mM NaCl, 1 mM EGTA, 1 mM EDTA, 10 mM Tris-HCl, pH 7.4, and protease inhibitors) at 4°C for 10 min, then centrifuged at 10000 *g *for 10 min at 4°C. Proteins were loaded onto a 12% SDS-polyacrylamide gels and transferred to nitrocellulose membranes. After blocking in 5% non-fat dried milk at room temperature for 30 min, membranes were probed with rabbit polyclonal anti-AIF antibody, followed by horseradish peroxidase-conjugated secondary antibodies. Bands were visualized using the ECL detection system (Amersham, Buckinghamshire, UK).

AIF nuclear translocation was further confirmed by immunofluorescence analysis. Cells were cultured on glass coverslips and treated with silibinin. Cells were washed twice with PBS, fixed with 4% paraformadehyde in PBS for 10 min, permeabilized with 0.5% Triton X-100 in PBS for 10 min. After washing twice with PBS, cells were blocked with 8% BSA in Tris-buffered saline Triton X-100 (TBST). Cells were incubated with rabbit polyclonal anti-AIF overnight 4°C and washed twice with TBST. Cells were incubated with FITC-conjugated secondary antibody (Jackson ImmunoResearch Laboratories, PA, USA) for 1 h, and the nuclei were counterstained with propidium iodide to ascertain AIF unclear localization. Cell were washed twice and visualized by using the confocal microscope (Leica, Wetzlar, Germany).

### RNA interference (RNAi)

For AIF targeting, we used The BLOCK-iT™ Pol miR RNAi Expression Vector Kits (Invitrogen, Carlsbad, CA, USA) to facilitate the expression of microRNA (miRNA). miRNA sequences for AIF were designed using online software (BLOCK-iT RNAi Designer from Invitrogen). The target sequence was 5'-GTGCCTATGCCTACAAGACTA-3'. This single-stranded oligonucleotide generated a double-stranded oligonucleotide, which instructed into pcDNA™ 6.2-GW/EmGFP-miR vector. This vector contains EmGFP that allow identifying of the transfection efficiency using fluorescence microscopy. The construct pcDNA™ 6.2-GW/EmGFP-miR-LacZ was used as a control. Cells were transiently transfected with these plasmids using lipofectamine (Invitrogen).

### Statistical analysis

The data are expressed as means ± SEM and the difference between two groups was evaluated using Student's *t*-test. Multiple group comparison was done using one-way analysis of variance followed by the Tukey post hoc test. A probability level of 0.05 was used to establish significance.

## Results and Discussion

### Effect of calpain inhibitor on silibinin-induced cell death

**Figure 1:**
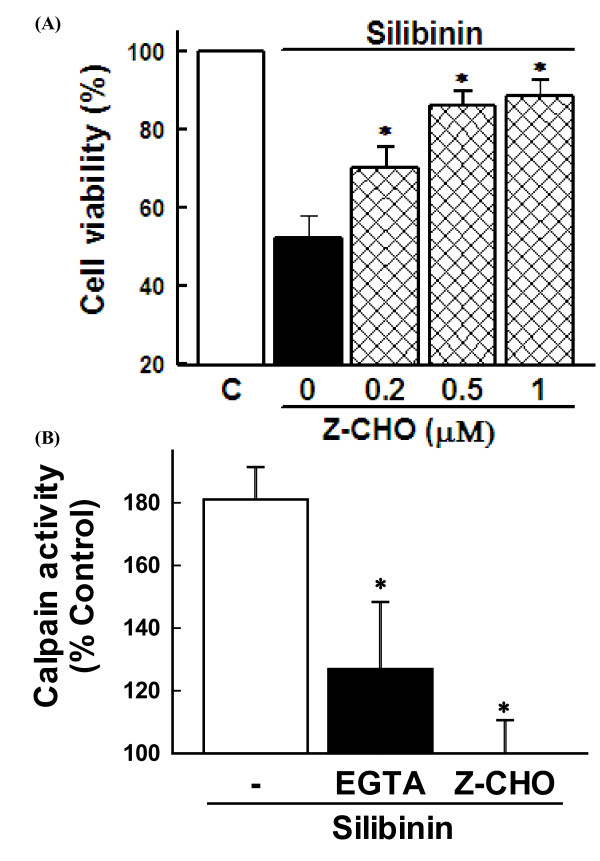
**Role of calpain in silibinin-induced cell death**. **(A) **Cells were exposed to 30 μM silibinin for 36 h in the presence of various concentrations of calpain inhibitor (Z-CHO). Cell viability was estimated by MTT assay. Data are mean ± SEM of four independent experiments performed in duplicate. *p < 0.05 compared with silibinin alone. **(B)** Cells were exposed to 30 μM silibinin for 24 h in the presence of 2 mM EGTA and 0.5 μM Z-CHO. Calpain activity was measured by calpain assay kit. Data are mean ± SEM of four independent experiments performed in duplicate. *p < 0.05 compared with silibinin alone.

Calpains are cytosolic Ca^**2+**^-activated neutral cysteine proteases and ubiquitously distributed in all animal cells, which play a critical role in regulating cell viability [11,12]. Accumulating evidence suggests that calpain activation may contribute to cell death in certain cell types including thymocytes, monocytes, cardiomyocytes, and neuronal cells [13]. Since our previous study showed that the calpain inhibitor Z-Leu-Leu-CHO at 0.5 μM significantly protected effectively against the silibinin-induced cell death [8], we observed in the present study the dose-dependency of the inhibitor effect. The results showed that the calpain inhibitor exerted protective effect against the silibinin-induced cell death in a dose-dependent manner with maximum potency at 0.5-1 μM (Figure 1A). Silibinin also induced calpain activation, which was blocked by EGTA and calpain inhibitor (Figure 1B). These results indicate that calpain activation plays a critical role in the silibinin-induced cell death in human glioma cells.

### Role of calpain and protein kinase C (PKC) activation in ROS generation and cell death induced by silibinin

**Figure 2:**
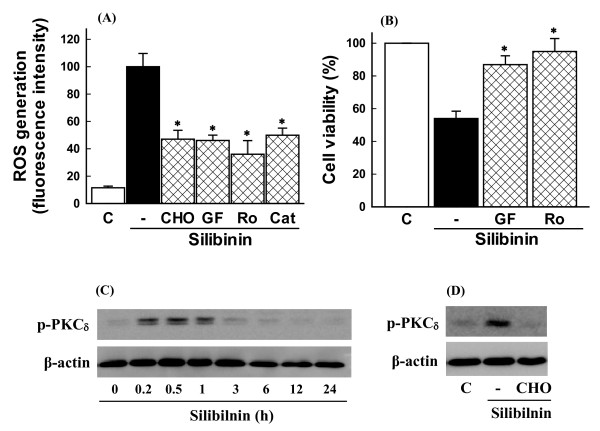
**Role of calpain and PKC in ROS generation and cell death induced by silibinin**. **(A)** Effect of inhibitors of calpain and PKC on silibinin-induced ROS generation. Cells were exposed to 30 μM silibinin in the presence or absence of 0.5 μM calpain inhibitor (CHO), 1 μM GF 109203X (GF), 1 μM rottlerin (Ro), and 800 units/ml catalase (Cat) and ROS generation was estimated by measuring changes in DCF fluorescence using FACS analysis. Data are mean ± SEM of five independent experiments performed in duplicate. *p < 0.05 compared with silibinin alone. **(B)** Effect of PKC inhibitors on silibinin-induced cell death. Cells were exposed to 30 μM silibinin in the presence or absence of 1 μM GF 109203X (GF) and 1 μM rottlerin (Ro) and cell viability was measured by MTT assay. Data are mean ± SEM of four independent experiments performed in duplicate. *p < 0.05 compared with silibinin alone. **(C)** Effect of silibinin on PKC_δ _activation. Cells were exposed to 30 μM silibinin for various times and PKC_δ _phosphorylation was estimated by Western blot analysis. **(D)** Effect of calpain inhibitor on PKC_δ _phosphorylation. Cells were exposed to 30 μM silibinin for 10 min in the presence or absence of 0.5 μM calpain inhibitor (CHO) and PKC_δ _phosphorylation was estimated by Western blot analysis.

The silibinin-induced cell death was associated with ROS generation mediated by intracellular Ca^2+ ^[8]. To determine therefore whether ROS production by silibinin is attributed to calpain activation, cells were exposed to silibinin in the presence of calpain inhibitor and ROS generation was measured. As shown in Figure 2A, the silibinin-induced ROS generation was blocked by the calpain inhibitor with potency similar to that of catalase.

PKCs are a family of serine/threonine kinases which are involved in tumor formation and progression [14]. PKC isoforms cooperate or exert opposite effects on the process of apoptosis [15,16]. PKC isoforms such as PKCα, ε, and ξ inhibit apoptosis, whereas PKC_δ _is involved in the process of apoptosis [16,17]. Although previous studies have shown that flavonoids can induce activation of PKC [18,19], it is unclear whether PKC is involved in the signaling cascade of silibinin-induced cell death. Although PKCs are activated by ROS [20,21], it has been reported that PKC activation can also cause ROS generation [22,23]. Therefore, we examined involvement of PKC in the silibinin-induced ROS generation. The general PKC inhibitor GF 109203X and the selective PKC_δ _inhibitor rottlerin blocked the ROS generation (Figure 2A). The silibinin-induced cell death was also prevented by the general PKC inhibitor GF 109203X and rottlerin (Figure 2B), indicating that silibinin induces ROS generation and cell death through PKC activation. We next examined whether silibinin induces PKC_δ _phosphorylation, an index of PKC_δ _activation. Silibinin induced a transient phosphorylation of PKC_δ _after 10 min of treatment, which was inhibited by treatment of calpain inhibitor (Figure 2C and 2D), suggesting that PKC_δ _may be a downstream of calpain in the silibinin-induced cell death. Similar results are reported in human U-937 leukemia cells in which the flavonoid wogonin induces cell arrest through PKC_δ _activation [18].

### Role of Bax expression and mitochondria in silibinin-induced cell death

**Figure 3:**
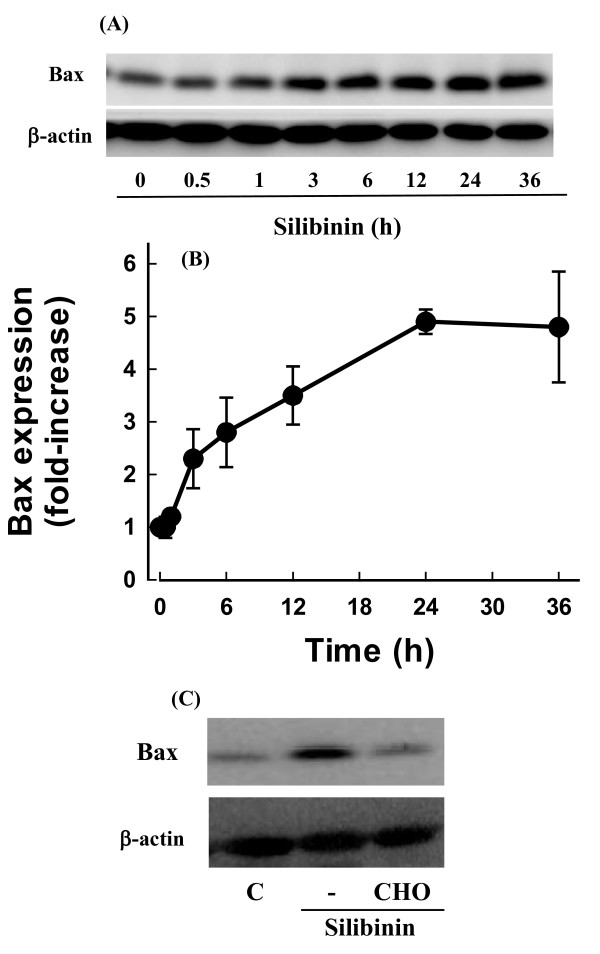
**Effect of silibinin on Bax expression**. Cells were exposed to 30 μM silibinin for various times and Bax expression was estimated by Western blot analysis. Representative **(**A**) **and quantitative **(B)** results of four independent experiments. **(**C**) **Cells were exposed to 30 μM silibinin for 24 h in the presence or absence of 0.5 μM calpain inhibitor (CHO) and Bax expression was estimated by Western blot analysis.

Since numerous death signals converge on mitochondria through the activation of pro-apoptotic members of the Bcl-2 family such as Bax [24], calpain activation may induce the silibinin-induced cell death through a Bax-dependent pathway. To test this possibility, the effect of silibinin on Bax expression was examined. Silibinin increased Bax expression after 3 h of treatment, which was blocked by the calpain inhibitor (Figure 3).

**Figure 4:**
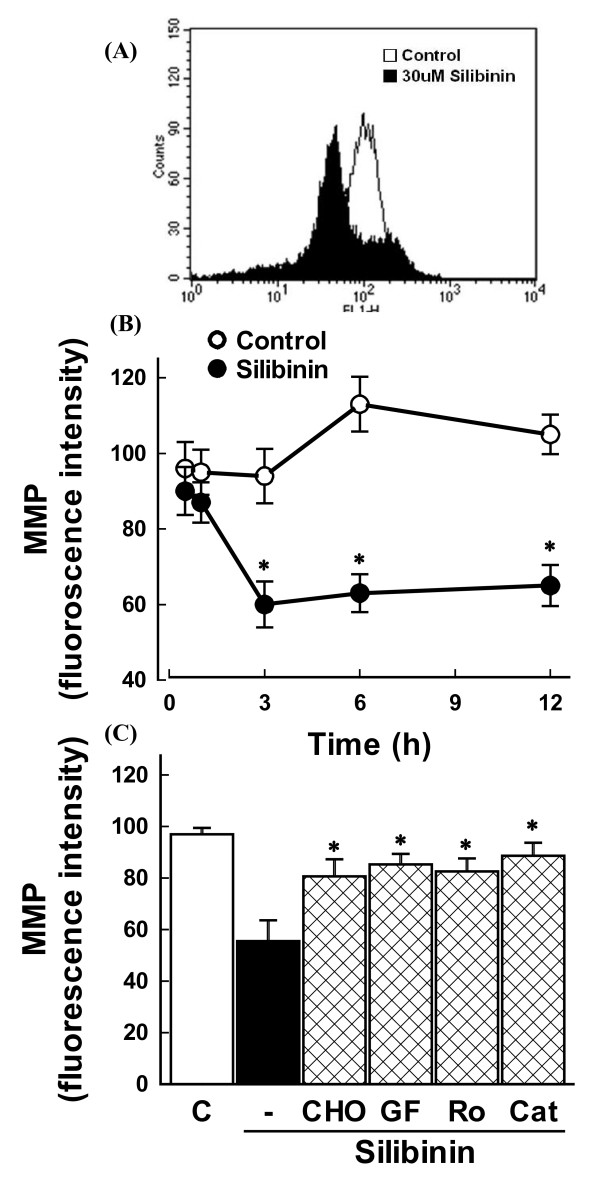
**Effect of silibinin on mitochondrial membrane potential (MMP)**. Cells were exposed to 30 μM silibinin for 6 h **(A) **and various times **(B)**. The MMP was estimated by the uptake of a membrane potential-sensitive fluorescence dye DiCO_6_(3). The fluorescence intensity was analyzed using FACS analysis. Data in (B) are mean ± SEM of three independent experiments performed in duplicate. *p < 0.05 compared with control. (C) Effect of inhibitors of calpain and PKC and antioxidant on silibinin-induced disruption of MMP. Cells were exposed to 30 μM silibinin for 6 h in the presence or absence of 0.5 μM calpain inhibitor (CHO), 1 μM GF 109203X (GF), 1 μM rottlerin (Ro), and 800 units/ml catalase (Cat). The MMP was measured as described above. Data are mean ± SEM of four independent experiments performed in duplicate. *p < 0.05 compared with silibinin alone.

The increase in Bax expression may cause disruption of △ψ_m _to induce cell death. To test the possibility, cells were exposed to silibinin and the △ψ_m _was measured using the fluorescence dye. After silibinin treatment, disruption of △ψ_m _was observed as evidenced by an increase in the proportion of cells with lower fluorescence intensity (Figure 4A). The reduction in △ψ_m _was observed after 3 h of silibinin treatment and remained unchanged even after 12 h (Figure 4B).

Disruption of △ψ_m _by silibinin may be associated with ROS generation. To test the possibility, cells were exposed to silibinin in the presence of the antioxidant catalase and △ψ_m _was measured. Figure 4C shows that the silibinin-induced reduction in △ψ_m _was blocked by catalase, suggesting that the △ψ_m _disruption by silibinin is mediated by ROS generation.

As shown above, since the silibinin-induced ROS generation was blocked by inhibitors of calpain and PKC, the silibinin-induced disruption of △ψ_m _would be prevented by these inhibitors. As expected, the reduction in △ψ_m _was blocked by Z-Leu-Leu-CHO, GF 109203X, and rottlerin, with similar potency to that by catalase (Figure 4C).

### Role of AIF nuclear translocation in silibinin-induced cell death

**Figure 5:**
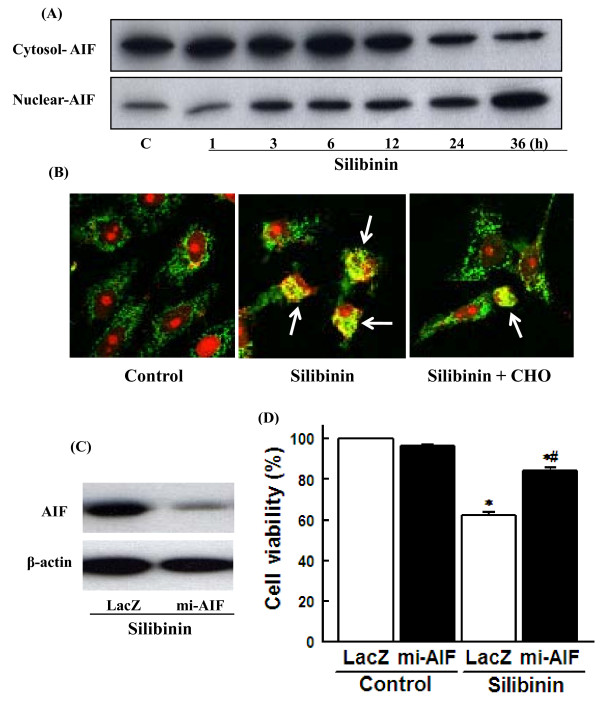
**Role of AIF nuclear translocation in silibinin-induced cell death**. **(**A**)** Cells were exposed to with 30 μM silibinin for various times and cytosolic and nuclear fractions were prepared. AIF expression was estimated by Western blot using antibodies specific against AIF. **(**B**) **Cells were exposed to 30 μM silibinin for 36 h in the presence or absence of 0.5 μM calpain inhibitor (CHO). AIF nuclear translocation was estimated by immunofluorescence using antibody specific against AIF. Nuclei were counterstained with propidium iodide (PI). Images were captured by confocal microscope and presented. Arrows indicate AIF nuclear localization. **(C) **Cells were transfected with mipcDNA vector for LacZ or AIF micro-RNA (mi-AIF). The expression levels of AIF were determined by Western blotting. **(D) **Cells transfected with LacZ or mi-AIF were exposed to 30 μM silibinin for 36 h and cell viability was estimated by MTT assay. Data are mean ± SEM of four independent experiments performed in duplicate. *p < 0.05 compared with LacZ control; #p < 0.05 compared with LacZ silibinin.

The mitochondrial apoptotic pathway is initiated by the cytosolic release of mitochondrial intermembrane space proteins that can trigger either caspase-activation or caspase-independent apoptotic pathways [25,26]. Mitochondrial proteins that cause caspase-dependent cell death include cytochrome c which triggers caspase-9 activation through Apaf-1. The activated caspase-9 then activates the downstream caspase-3 [26-28]. Mitochondria have also been reported to contain AIF, which can cleave directly DNA and intracellular substrates when released into the cytosol. During apoptosis, AIF translocates into the nucleus where it causes oligonucleosomal DNA fragmentation [29,30]. The present study showed that silibinin causes AIF nuclear translocation, which was inhibited by the calpain inhibitor (Figure 5A and 5B). To determine if silibinin induced cell death through AIF nuclear translocation, effect of silibinin on the cell death in cells transfected with AIF mi-RNA was measured. Transfection of AIF mi-RNA was decreased AIF protein levels (Figure 5C) and effectively prevented the silibinin-induced cell death (Figure 5D). These data suggest that calpain activation induces AIF-dependent cell death in silibinin-treated cells. This is the first report showing involvement of calpain-dependent AIF nuclear translocation in the silibinin-induced glioma cell death.

## Conclusion

The present study demonstrated that silibinin induces apoptosis through AIF nuclear translocation mediated by a calpain-dependent pathway in U87MG human glioma cells. This pathway involves PKC activation and ROS generation. These data suggest that silibinin may be considered a potential candidate in prevention and treatment of human malignant gliomas.

## List of abbreviations

AIF: apoptosis-inducing factor; DCF: 2',7'-dichlorofluorescein; DCFH-DA: 2',7'-dichlorofluorescein diacetate; DiOC_6_(3): 3,3'-dihexyloxacarbocyamide; MTT: 3-[4,5-dimethylthiazol-2-yl]-2,5-diphenyltetrazolium bromide; PBS: phosphate buffer solution; PKC: protein kinase C; ROS: reactive oxygen species; △ψ_m_: mitochondrial membrane potential.

## Competing interests

The authors declare that they have no competing interests.

## Authors' contributions

JJ carried out cell viability and apoptosis assay, participated in drafted the manuscript. WS and TK carried out mitochondrial membrane potential, ROS generation, and statistical analyses. CK and YK carried out Western blot, calpain activity, and AIF nuclear translocation. KK and JK participated in experiment design and the draft preparation. All authors read and approved the final manuscript.
